# Poisoning by Aconite

**Published:** 1855-12

**Authors:** 


Poisoning by Aconite.—Bappoo Kishnura, a Hindoo priest, aged 50, was admitted into the Jamsetjee Jejeebhoy hospital, in the afternoon of the 4th of August, 1854, under the care of J. Peet, Esq., Assistant-Surgeon. For 24 days he had been taking, daily, 15 grs. of a native drug called 'bishnak,' (a root of the aconitum ferox) as a remedy for leprosy, from which he had suffered for several years. Till the morning of the day of his admission he had been using the black variety of the drug, but at the recommendation of a friend he substituted the white variety. The dose was 15 grs., and was taken in the morning at 10 o'clock. He soon afterwards began to feel uneasy, had a disagreeable burning sensation in the mouth and fauces, a sense of formication, and some confusion of mind. A friend recommended milk, of which he drank a large quantity. Soon afterwards he vomited freely; and about an hour after the vomiting came, and about four hours from the taking of the drug, he was brought to the hospital. He was then sensible, had a stupid expression of the face, and walked with an unsteady gait, like a drunken person. He complained of heat and burning in the throat, some confusion of mind, and a sense of formication over the surface generally. There was almost constant vomiting; pulse feeble and rapid; skin coldish, and covered with moisture; pupils natural. A sinapism was applied to the epigastrium, and stimulants of ammonia were administered. The vomiting continued till 12 o'clock. Ultimately the animal warmth returned, the pulse rose, the vomiting ceased. He went home in the course of the day.—*Transactions of the Medical and Physical Society of Bombay*, 1855.

